# Circulating mitochondrial DNA: New indices of type 2 diabetes-related cognitive impairment in Mexican Americans

**DOI:** 10.1371/journal.pone.0213527

**Published:** 2019-03-12

**Authors:** Talisa Silzer, Robert Barber, Jie Sun, Gita Pathak, Leigh Johnson, Sid O’Bryant, Nicole Phillips

**Affiliations:** 1 Department of Microbiology, Immunology, Genetics, University of North Texas Health Science Center, Fort Worth, Texas, United States of America; 2 Department of Pharmacology and Neuroscience, University of North Texas Health Science Center, Fort Worth, Texas, United States of America; 3 Institute for Healthy Aging, University of North Texas Health Science Center, Fort Worth, Texas, United States of America; Duke University, UNITED STATES

## Abstract

Mitochondrial function has been implicated and studied in numerous complex age-related diseases. Understanding the potential role of mitochondria in disease pathophysiology is of importance due to the rise in prevalence of complex age-related diseases, such as type 2 diabetes (T2D) and Alzheimer’s disease (AD). These two diseases specifically share common pathophysiological characteristics which potentially point to a common root cause or factors for disease exacerbation. Studying the shared phenomena in Mexican Americans is of particular importance due to the disproportionate prevalence of both T2D and AD in this population. Here, we assessed the potential role of mitochondria in T2D and cognitive impairment (CI) in a Mexican American cohort by analyzing blood-based indices of mitochondrial DNA copy number (mtDNA_CN_) and cell-free mitochondrial DNA (CFmtDNA). These mitochondrial metrics were also analyzed for correlation with relevant neuropsychological variables and physiological data collected as indicators of disease and/or disease progression. We found mtDNA_CN_ to be significantly decreased in individuals with CI, while CFmtDNA was significantly elevated in T2D; further, CFmtDNA elevation was significantly exacerbated in individuals with both diseases. MtDNA_CN_ was found to negatively correlate with age and fatty acid binding protein concentration, while positively correlating with CFmtDNA as well as CERAD total recall score. Candidate gene SNP-set analysis was performed on genes previously implicated in maintenance and control of mitochondrial dynamics to determine if nuclear variants may account for variability in mtDNA_CN_. The results point to a single significant locus, in the *LRRK2/MUC19* region, encoding leucine rich repeat kinase 2 and mucin 19. This locus has been previously implicated in Parkinson’s disease, among others; rs7302859 was the driver SNP. These combined findings further indicate that mitochondrial dysfunction (as assessed by proxy via mtDNA_CN_) is intimately linked to both T2D and CI phenotypes as well as aging.

## Introduction

### Type 2 diabetes, Alzheimer’s disease and Mexican Americans

As the global elderly population continues to grow, a rapid increase is predicted for the prevalence of complex age-associated diseases such as cardiovascular disease, cancer, metabolic disorders and neurodegenerative diseases [AAAS, 2008; CDC, 2015]. Alzheimer’s disease (AD), the most common form of dementia, is listed as the 6^th^ leading cause of death within the U.S. [NIA Alzheimer’s Disease Fact Sheet, 2016] and affects over 5.5 million people in America alone. AD is a multifactorial disease with both genetic and environmental components and several comorbidities are capable of increasing an individual’s risk for developing AD later in life.

Type 2 diabetes (T2D) increases risk for a variety of diseases (*e*.*g*. cardiovascular disease, cerebrovascular disease and neuropathy) [1], including AD, which may be due common genetic-based etiology [2]. T2D confers a 2-fold increase in risk for AD and has been associated with development of more severe forms of cognitive impairment [3]. Over 30.3 million individuals in the United States have T2D and approximately 84.1 million are living in a pre-diabetic condition [CDC Fact Sheet, 2017]. Current prevalence of T2D may point to a potential increase in AD prevalence in the future.

Of particular research interest are populations whose prevalence of T2D and dementia are disproportionately high such as Mexican Americans. Hispanic Americans represent a growing sub-population within the United States comprising 17.6% of the total U.S. population, with approximately 63% being of Mexican origin [4]. Mexican American individuals are twice as likely to develop T2D throughout their lifetime, which may lead to the development of other comorbid conditions such as cardiovascular disease, cancer, stroke and Alzheimer’s disease [5]. In fact, the Sacramento Area Latino Study on Aging in 2003, found 43% of Mexican American subjects with dementia to also suffer from T2D [6]. AD is the most prevalent form of dementia and Mexican Americans generally suffer from earlier onset and more severe forms of AD [7]. Economic status and years of education negatively correlate with risk for dementia, where individuals with fewer years of education or of lower economic status are at increased risk, which may be due to poor heath, a characteristic commonly associated with lower economic status [6].

Interestingly, the genetic involvement in AD risk for Mexican Americans is seemingly quite different than that of Caucasian populations. In Caucasians, variants of the gene encoding the lipoprotein APOE (specifically the ε4 allele) have been positively associated with risk for AD. Although the risk associated with the APOE ε4 allele is thought to be similar to that of non-Hispanic individuals, the allele is much less frequent in Mexican American populations [6, 8]. Nevertheless, even in the absence of the ε4 allele, Mexican American individuals have twice the risk for developing AD when compared to non-Hispanic individuals [6]. In addition, Mexican Americans with AD appear to have a more metabolic phenotype. This is demonstrated by the distinct biomarker profile which is predictive of AD in Mexican Americans and consists of proteins associated with obesity, insulin resistance, T2D and metabolic syndrome (*e*.*g*., FABP, GLP1, IGFBP2 [9]). The predictive biomarker profile for Caucasians is more inflammatory in nature by comparison.

Due to the disproportionate prevalence within Mexican American populations, as well as financial and psychological burdens associated with these diseases, it is important to further our understanding of the pathophysiological mechanisms leading to these comorbid conditions. Of the various hypotheses and theories, many researchers (including our group) have shown evidence that mitochondria may be at the root of the pathophysiology due to its key involvement in a variety of cellular processes (*e*.*g*., apoptosis, calcium homeostasis and oxidative stress signaling). All of these processes have been shown to be impaired and/or altered in both T2D and AD [10–13].

### Mitochondrial biology

Mitochondria have unique roles in a variety of signaling cascades involved in many cellular processes, with their primary function being to generate ATP through electron transport chain (ETC) and oxidative phosphorylation; however, this process is also accompanied by generation and release of reactive oxygen and nitrogen species (ROS, RNS) including compounds such as hydrogen peroxide (H_2_O_2_), hypochlorous acid (HOCl), hydroxyl radical (OH^-^) and superoxide anion (O2^-^) [14]. The close proximity to accumulating oxidative species leave mitochondria, specifically mitochondrial DNA (mtDNA), susceptible to damage. Due to their intricate ties to a variety of cellular functions which have been shown to decline in aging and disease, different mitochondrial-based theories of aging and age-related disease have arisen (*e*.*g*. mitochondrial theory of aging.).

### Mitochondrial theory of aging and mitochondrial DNA

The free radical theory of aging, sometimes referred to the mitochondrial theory of aging, was first developed by Denham Harmon and suggests that single processes such as free radical reactions can be modified by both genetic and environmental factors to increase the probability of human disease and/or death [15]. Due to its close proximity to the ETC, mtDNA is particularly susceptible to damage which is problematic since mtDNA encodes some of the proteins required for oxidative phosphorylation (with the rest being encoded by nuclear DNA (nDNA)), and damage to mtDNA may lead to overall mitochondrial dysfunction. Although mitochondrial genomes are present in multiple copies per cell (depending on the cell/tissue type), they naturally accumulate mutations over time and lack many of the mechanisms in place for nDNA repair. Mitochondrial dysfunction, mtDNA mutations, and decreased mtDNA copy number (mtDNA_CN_) and content have been associated with aging and various disease states. Although copy number is under strict regulation, the mechanisms of this regulation remain relatively unclear [16]. Mitochondrial dynamics including processes such as fission, fusion and mitophagy, work to maintain copy number and the quality of the overall mitochondrial population. Fusion has been proposed as a mechanism for regulating mtDNA populations, allowing for mixing of heteroplasmic genomes; fission has been suggested to regulate mitochondrial quality and biogenesis and is capable of triggering mitophagy (targeted removal of mitochondria) in mitochondrial organelles that are malfunctioning or that contain detrimental mutations [17].

Mitochondrial DNA copy number is used as a proxy for mitochondrial biogenesis and has been proposed as a biomarker for a variety of diseases [18–22]. For example, the Mitochondrial Cascade Hypothesis of AD developed by Swerdlow and colleagues, position mitochondria at the root of disease onset, suggesting that mitochondrial function declines over time with no phenotypic consequence until reaching a particular threshold at which the pathophysiological hallmarks of AD ensue [23]. Under this hypothesis, mtDNA_CN_ as well as other more direct assessments of mitochondrial function, may serve as a means of quantifying the degree of mitochondrial dysfunction in aging and age-related disease.

### “Inflamm-aging” and cell-free mitochondrial DNA

Another competing theory, although not mutually exclusive, is “inflamm-aging”. This term coined by Franceschi and colleagues attributes aging (and age-related diseases) to progressive and continuous up-regulation of systemic inflammatory responses, irrespective of pathogenic infection [24]. It is thought that this “inflamm-aging” is due to attenuation or exhaustion of adaptive immune responses; in particular, constant stimulation of macrophages over time may drive an individual into a pro-inflammatory state [24]. Mitochondria play a key role in inflammatory responses by participating in metabolism and cell death signaling. In addition, under conditions of high stress, mitochondria can become damaged resulting in extrusion of mitochondrial DNA within mitochondrial derived vesicles (MDVs) via an exosomal-type mechanism [25]. Cell-free mitochondrial DNA (CFmtDNA) is then capable of acting as a damage associated molecular pattern (DAMP), most likely due to the similarity to its bacterial ancestors (*e*.*g*. circular, lack of histones). CFmtDNA has been found to activate a variety of pro-inflammatory responses involving macrophages, NLRP3 inflammasomes and neutrophils (via TLR9 receptors), as well as other downstream pro-inflammatory signaling proteins such as TNFα and NFκB (indirectly) [26, 27]. CFmtDNA has been broadly studied as a biomarker for systemic inflammation in a number of diseases whose pathophysiologies contain inflammatory components [28–30] and is therefore a relevant phenotype to T2D and AD, both of which have tissue specific and systemic inflammatory components.

### Study objective

In this study we investigate the potential role of mitochondria in the shared pathophysiology of T2D and AD by quantifying cell-free mtDNA and mtDNA copy number as means of gauging levels of systemic inflammation and mitochondrial biogenesis (respectively) in individuals of varying conditions. In addition, we explore whether mitochondrial indices such as copy number have significant relationships with different physiological and neuropsychological variables. Lastly, we examine the potential significant associations between mitochondrial copy number and variants in candidate genes whose products are involved in mitochondrial-related processes such as biogenesis, fusion, fission and mitophagy, to gain further insight into the regulation of copy number.

## Methods

### Samples and cohort design

This study was approved under the University of North Texas Health Science Center IRB #2012–083; subjects provided written consent for participation. Peripheral blood of fasted-state female Mexican American subjects (n = 46, self-reported race/ethnicity; Table 1) enrolled in the Health & Aging Brains of Latino Elders (HABLE) study was analyzed in this work. Sample size and subject selection for this study were out of convenience due to difficulty of pair-wise matching. This study was also limited to female subjects in order to avoid confounding variables such as sex. Other studies by our lab have demonstrated a sex difference in specific mitochondrial indices specifically in a Caucasian population [31]. Blood samples were handled in accordance to Institutional Biosafety Committee approved protocol IBC-2018-0078. Twenty-three T2D affected subjects (self-reported, HbA1c >6%) were chosen, exhibiting a range of cognitive function, from normal to probable Alzheimer’s disease. Twenty-three age- and cognition-matched non-diabetic control subjects were then identified to complete the study population (n = 46). A single pair of individuals with probable AD were imperfectly matched (69 vs. 70 years old). All other pairs were matched to the year. Matching across the two groups on age and cognition was performed to account for known associations between T2D and age, as well as cognition and T2D. Approximately 96% of diabetic subjects were taking oral mediation (unspecified) to control diabetes at the time of sampling; 26% of subjects were taking insulin injections in addition to oral medications. The average duration of T2D (age at sampling minus age of onset) was 11.52 years (± 6.28 SD). The average HbA1c percentage for the diabetic and non-diabetic groups was 8.25 (± 1.52 SD) and 5.63 (± 0.22 SD), respectively. Cognitive status was categorized into normal, mild cognitive impairment (MCI) or probable Alzheimer’s disease (AD) sub-groups based on the results of a battery of neuropsychological tests (*e*.*g*. CDR sum of boxes, MMSE, clock drawing). Due to small sample size, MCI and AD subjects were clustered together into a cognitively impaired (CI) group when conducting statistical analyses. Descriptive statistics of the cohort can be found in Table 1, where the group is divided in three ways: (1) by diabetic status, (2) by cognitive status, and (3) by the intersection of diabetes and cognitive impairment.

**Table 1:** Descriptive statistics of female, Mexican American (self-described) subject cohort. Variable sample sizes for each category are due to missing subject responses. CDR, clinical dementia rating (ranges from 0–18, negatively correlated with cognitive function); MMSE, mini mental state exam (ranges from 0–30, positively correlated with cognitive function).

		Age (years)	Education (years)	Annual household income	Body Mass Index	Blood—Glucose (md/dL)	CDR: Sum of Boxes	MMSE
Diabetes: NO	N	23	23	21	22	23	23	23
	Mean	60.43	9.13	$28,790.48	33.08	94.22	.87	26.04
	SEM	0.95	1.10	$5,230.05	1.89	2.35	0.73	1.05
Diabetes: YES	N	23	23	23	23	23	23	23
	Mean	61.00	5.91	$17,648.61	34.70	160.57	0.46	25.87
	SEM	1.11	0.72	$2,490.24	1.46	11.14	0.21	0.56
*p*-value, t-test		0.70200	0.19000	0.64000	0.50000	0.00001	0.53900	0.88500
Cognitive Impairment: NO	N	35	35	34	34	35	35	35
	Mean	59.17	8.09	$24,126.47	34.24	126.60	0.00	27.14
	SEM	0.66	0.79	$3,569.65	1.38	8.34	0.00	0.346
Cognitive Impairment: YES	N	11	11	10	11	11	11	11
	Mean	65.64	5.73	$19,021.80	32.90	129.91	2.77	22.18
	SEM	1.42	1.36	$4,069.84	2.36	17.40	1.46	1.83
*p*-value, t-test		0.00004	0.14900	0.47800	0.63300	0.85300	0.08700	0.02300
Diabetes: NO; Cognitive Impairment: NO	N	18	18	17	17	18	18	18
	Mean	59.22	9.89	$29,176.47	33.21	94.44	0.00	27.67
	SEM	0.92	1.17	$6,310.14	2.38	2.86	0.00	0.40
Diabetes: NO; Cognitive Impairment: YES	N	5	5	4	5	5	5	5
	Mean	64.80	6.40	$27,150.00	32.64	93.40	4.00	20.20
	SEM	1.96	2.71	$7,329.56	2.36	3.75	3.25	3.81
Diabetes: YES; Cognitive Impairment: NO	N	17	17	17	17	17	17	17
	Mean	59.12	6.18	$19,076.47	35.26	160.65	0.00	26.59
	SEM	0.97	0.88	$3,091.37	1.43	12.42	0.00	0.56
Diabetes: YES; Cognitive Impairment: YES	N	6	6	6	6	6	6	6
	Mean	66.33	5.17	$13,603.00	33.12	160.33	1.75	23.83
	SEM	2.16	1.33	$3,676.98	4.08	26.33	0.56	1.19
*p*-value, Kruskal-Wallis		0.006	0.046	0.317	0.394	<0.0001	<0.0001	0.003

### Quantification of circulating cell-free mitochondrial DNA from peripheral blood plasma

DNA was extracted from 200 μL aliquots of peripheral blood plasma using the Prepfiler Express kit (Applied Biosystems, Foster City, CA) on the Automate Express robot. MtDNA was quantified using a Taqman qPCR assay [32] on a 7500 real-time PCR instrument (rt-PCR) (Applied Biosystems). An eight-point standard curve was run in duplicate for absolute quantification and to assess pipetting reproducibility. Mean Ct and resulting quantification results from the duplicate reactions were used for subsequent analyses. The accuracy of the standard curve was assessed by considering the y-intercept, R^2^ (>0.98) and slope values. Differences in cycle threshold (Ct) were compared to the standard curve, and all samples were analyzed on the same plate/run.

### Quantification of mitochondrial DNA copy number from peripheral blood buffy coat

DNA was extracted from peripheral blood buffy coat using the Prepfiler BTA Express kit (Applied Biosystems), on the Automate Express robot. Nuclear DNA and mtDNA were quantified using the Quantifiler Duo (Applied Biosystems) and Taqman qPCR assay [32] respectively, on a 7500 rt-PCR instrument (Applied Biosystems). An eight-point standard curve was run in duplicate for absolute quantification and to assess pipetting reproducibility. Mean Ct and resulting quantification results from the duplicate reactions were used for subsequent analyses. All samples were analyzed on the same plate/run. MtDNA copy number was derived from the ratio of nDNA: mtDNA (as described in [33]).

### Statistical analyses

#### Mitochondrial DNA

Statistical analyses were performed using Microsoft Excel and IBM SPSS V24.0 software. Power analyses for detecting differences in CFmtDNA between T2D- and T2D+ groups were conducted based on data provided by Liu. *et al*. [30] (for expected group means) and Pinti *et al*. [34] (for expected group standard deviations). A sample size of 20 was expected to produce a power of 95%. Based on previous studies by our group, we expected to be underpowered for detection of differences in mtDNA_CN_. A Shapiro Wilk test revealed the mtDNA_CN_ data to be normally distributed, while the CFmtDNA data was not. Accordingly, a log_10_ transformation was performed on the CFmtDNA data to allow for use of parametric testing. Average mtDNA_CN_ and CFmtDNA levels for individuals with/without either condition (T2D or CI) were compared using independent samples *t*-tests. Pearson’s correlations were used to determine whether significant relationships existed between CFmtDNA and/or mtDNA_CN_ with age respectively. ANOVA with Tukey post hoc test was also performed to test for significant differences in mtDNA_CN_ and CFmtDNA levels between those who suffer from neither disease and those who suffer from either or both diseases. Further, a Shapiro-Wilk test was performed to determine normality of neuropsychological testing variables and biological data yielding both normal and abnormal data. Accordingly, Pearson’s correlation and Spearman’s Rho tests were used to determine the presence of any significant relationships between certain neuropsychological (*e*.*g*., CERAD, MMSE, CDR) or physiological variables (*e*.*g*., routine blood work measures, specific serum protein quantifications [9]) and mtDNA_CN_. Variables were selected based on biological relevance to (i) cognition and metabolic function, and (ii) the predictive blood-based test for AD in Mexican Americans. All results with a *p*-value <0.05 were considered to be suggestive (not corrected for multiple testing).

### Candidate gene association study

Forty of the 46 subjects were selected for genotyping; three normal controls (T2D-/CI-) and three T2D subjects (T2D+/CI-) were omitted from the analysis due to resource limitations (*i*.*e*., 8 samples per array). All samples were genotyped on the Infinium Omni2.5Exome-8 DNA microarray (Illumina, San Diego, CA), per manufacturer’s guidelines. The array genotypes 2,608,742 markers including common, rare, and enriched exonic SNPs. Principle component analysis using smartpca [35] was first conducted to determine if an appreciable degree of heterogeneity was present in the sample set. Linkage disequilibrium-based pruning of the entire SNP-set using protocols in PLINK (—indep-pairwise 50 5 0.2) followed by SNP extraction from the full data set generated a dataset with 266,717 SNPs in linkage equilibrium. Three individuals were identified as outliers and were removed prior to subsequent set-based association tests.

Candidate genes were selected by reviewing recent literature and identifying genes involved in mitochondrial dynamics (*e*.*g*. fission and fusion), mitochondrial biogenesis, and mitophagy [36–41]. SNPs were mapped to genes, and a SNP-set was created for each candidate gene (intergenic SNPs ± 300kb window to include potential distal, cis-acting variants).

Permutation, set-based testing methods were implemented in PLINK [42] using the following parameters:—mind 0.1,—geno 0.05,—hwe 0.001,—maf 0.05,—mperm 10000,—set-r2 0.8,—set-p 0.05,—set-max 10. General Linear Model with covariate analysis of age and the first two principle components (EV1 and EV2) was applied to test for single SNP based associations with mtDNA_CN_.

Local Manhattan plots were generated using LocusZoom [43]. The accuracy of SNP clustering and genotype calling algorithms were verified by manually inspecting the clustering plots in GenomeStudio 2.0 (Illumina). Further, Q-Q plots of single SNP association results were checked for genomic inflation using the r package qqman [44]. LD calculations were derived from 1000 Genomes Nov. 2014 AMR (Admixed American) database.

## Results

### Cell-free mitochondrial DNA

**Fig 1:**
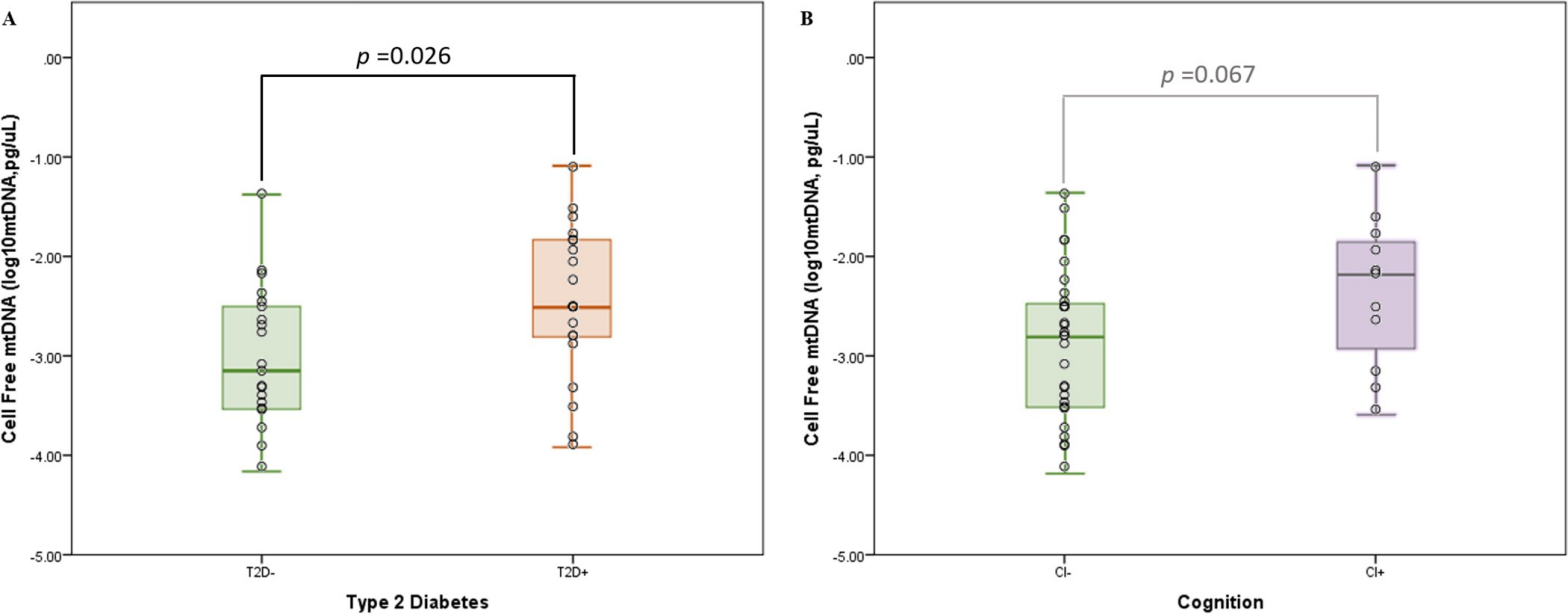
Average cell-free mitochondrial DNA levels stratified on diabetes and cognitive status. (A) Cell-free levels appear to be higher in individuals with diabetes (t-test p-value, 0.026) and those with cognitive impairment (B) (t-test p-value = 0.067, insignificant). A larger effect was observed for diabetic individuals. Note, age is matched between the T2D stratified analysis, but is not matched in the CI stratified analysis (i.e., CI+ subjects are older than CI-).

**Fig 2:**
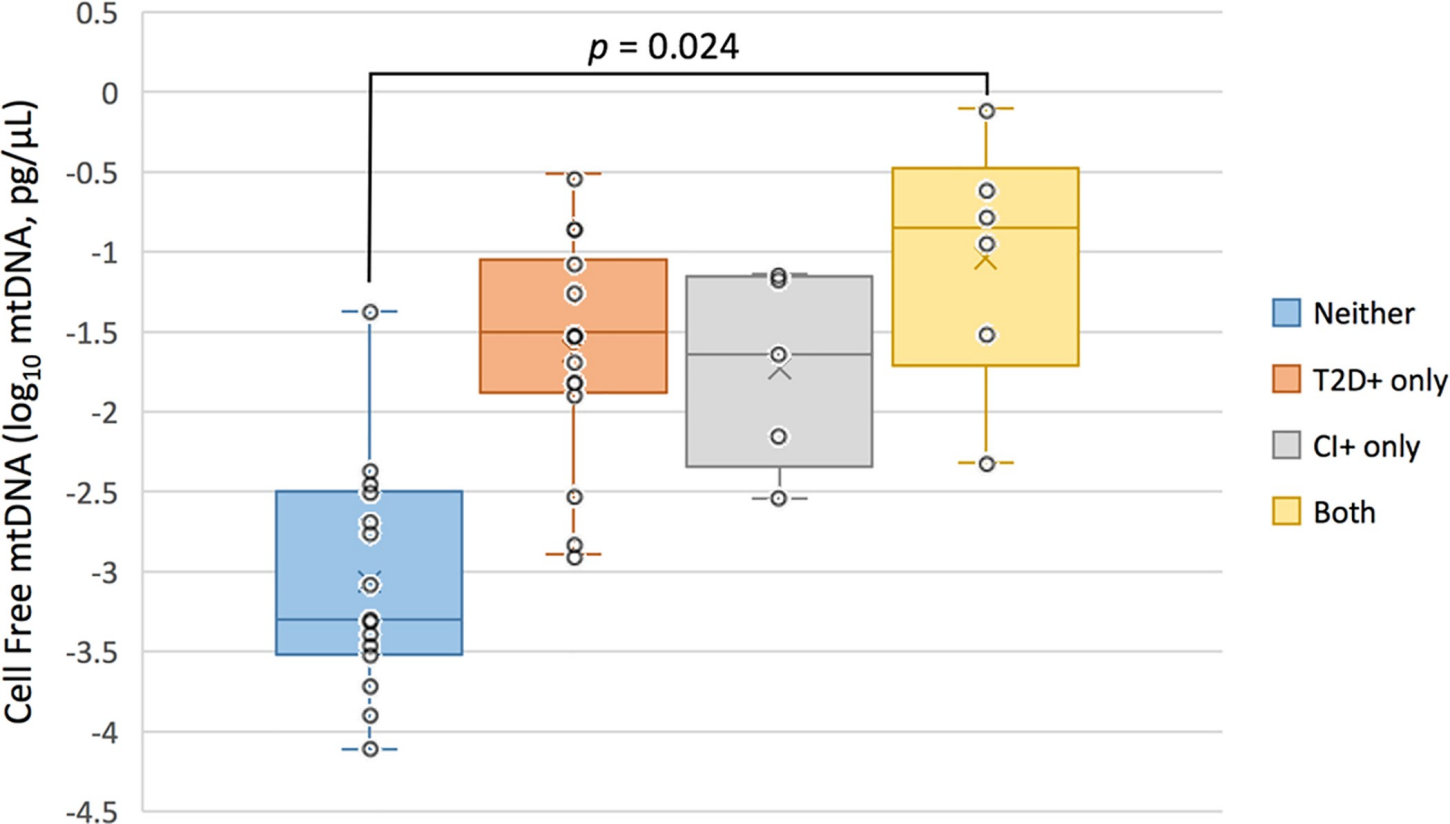
Average cell-free mitochondrial DNA levels stratified by condition. Cell-free mitochondrial DNA levels appear to be significantly higher in individuals suffering from both diseases (T2D and CI) when compared to controls (ANOVA, p-value 0.037, with significant Tukey post-hoc p-value = 0.024 as shown; respective Shapiro-Wilk test for normality, p-values = 0.521, 0.459, 0.443, 0.772).

**Fig 3:**
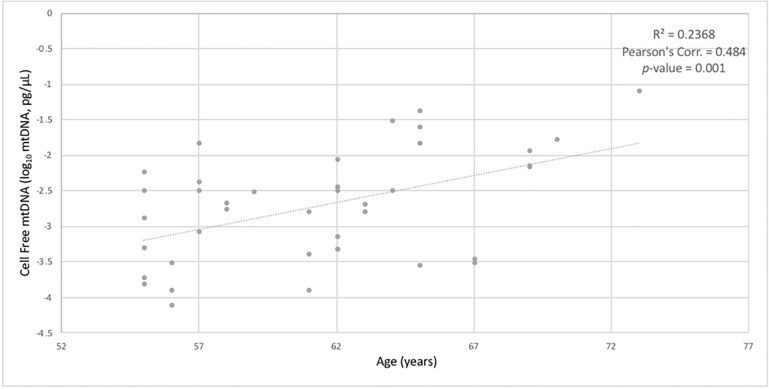
Correlation between cell-free mtDNA and age. Pearson’s correlations showing significant relationships between cell-free mitochondrial DNA levels and age (p-value = 0.001).

CFmtDNA levels were quantified from peripheral blood of each HABLE subject. When stratifying for T2D status, an independent *t*-test found diabetic individuals (238 copies, pg/μL) to have significantly higher CFmtDNA levels when compared to non-diabetic individuals (71 copies, pg/μL) (*p*-value = 0.026) (Fig 1A). However, an independent *t*-test showed an insignificant difference in CFmtDNA levels in cognitively impaired individuals (263 copies, pg/μL) when compared to cognitively intact individuals (85 copies, pg/μL) (*p*-value = 0.067) (Fig 1B). When stratifying for all four conditions, 1) neither disease, 2) T2D+ only, 3) CI+ only and 4) both diseases, ANOVA with Tukey post-hoc yielded a significant difference in CFmtDNA levels between controls and individuals suffering from both diseases (*p*-value = 0.024) (Fig 2). Pearson’s correlation revealed CFmtDNA levels to be significantly correlated with age (*p*-value = 0.001) (Fig 3).

### Mitochondrial DNA copy number

**Fig 4:**
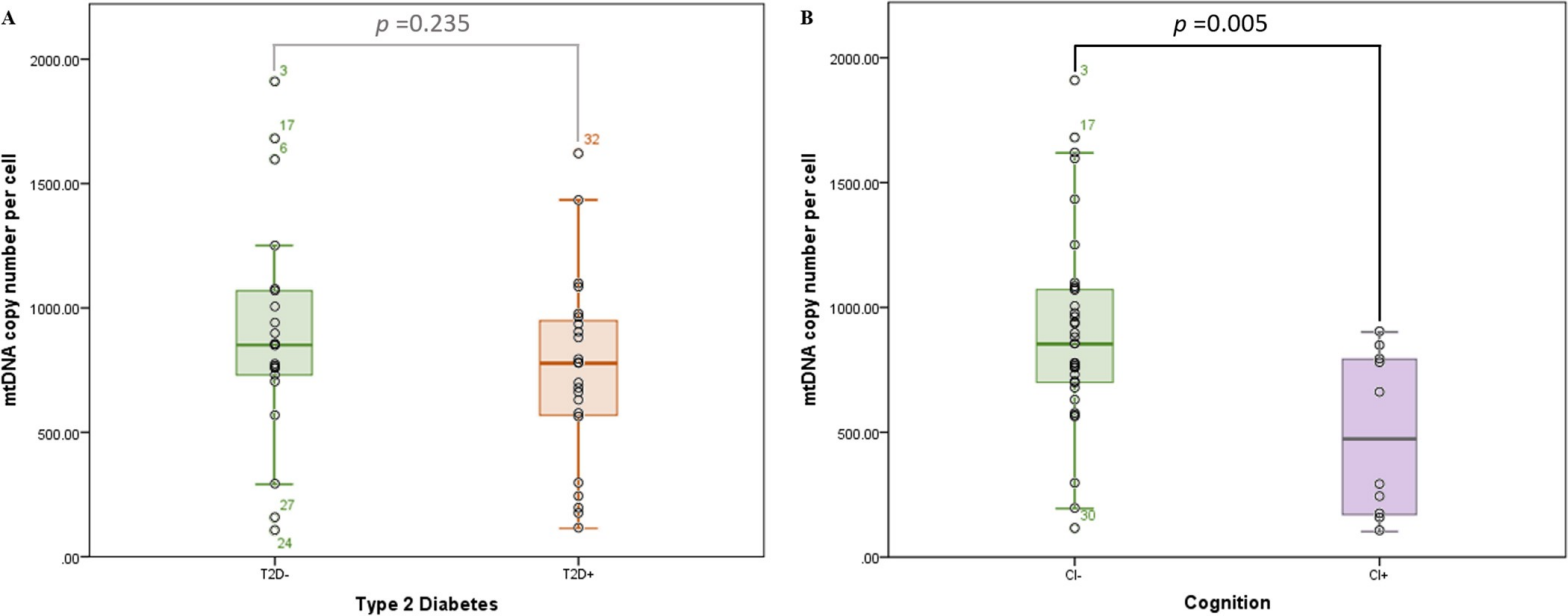
Average mitochondrial DNA copy number per cell stratified by diabetes and cognitive status. Copy number of mitochondrial genomes appears to be lower in individuals with (A) diabetes (t-test p-value = 0.235, insignificant) or, (B) cognitive impairment (t-test p-value = 0.005). A larger effect was observed for cognitively impaired individuals.

Mitochondrial DNA copy number was determined from peripheral blood of HABLE subjects. When stratifying for T2D status (T2D- (n = 23), T2D+ (n = 23)), an independent *t*-test showed diabetic individuals’ mtDNA_CN_ quantifications to be comparable to non-diabetic individuals (759 copies/cell and 920 copies/cell respectively) (*p*-value = 0.235) (Fig 4A). However, when stratifying for cognitive status (CI- (n = 35), CI+ (n = 11)), an independent *t*-test demonstrated mtDNA_CN_ to be significantly lower in individuals with cognitive impairment (either MCI or AD) (497 copies/cell) when compared to controls (804 copies/cell) (*p*-value = 0.005) (Fig 4B).

**Fig 5:**
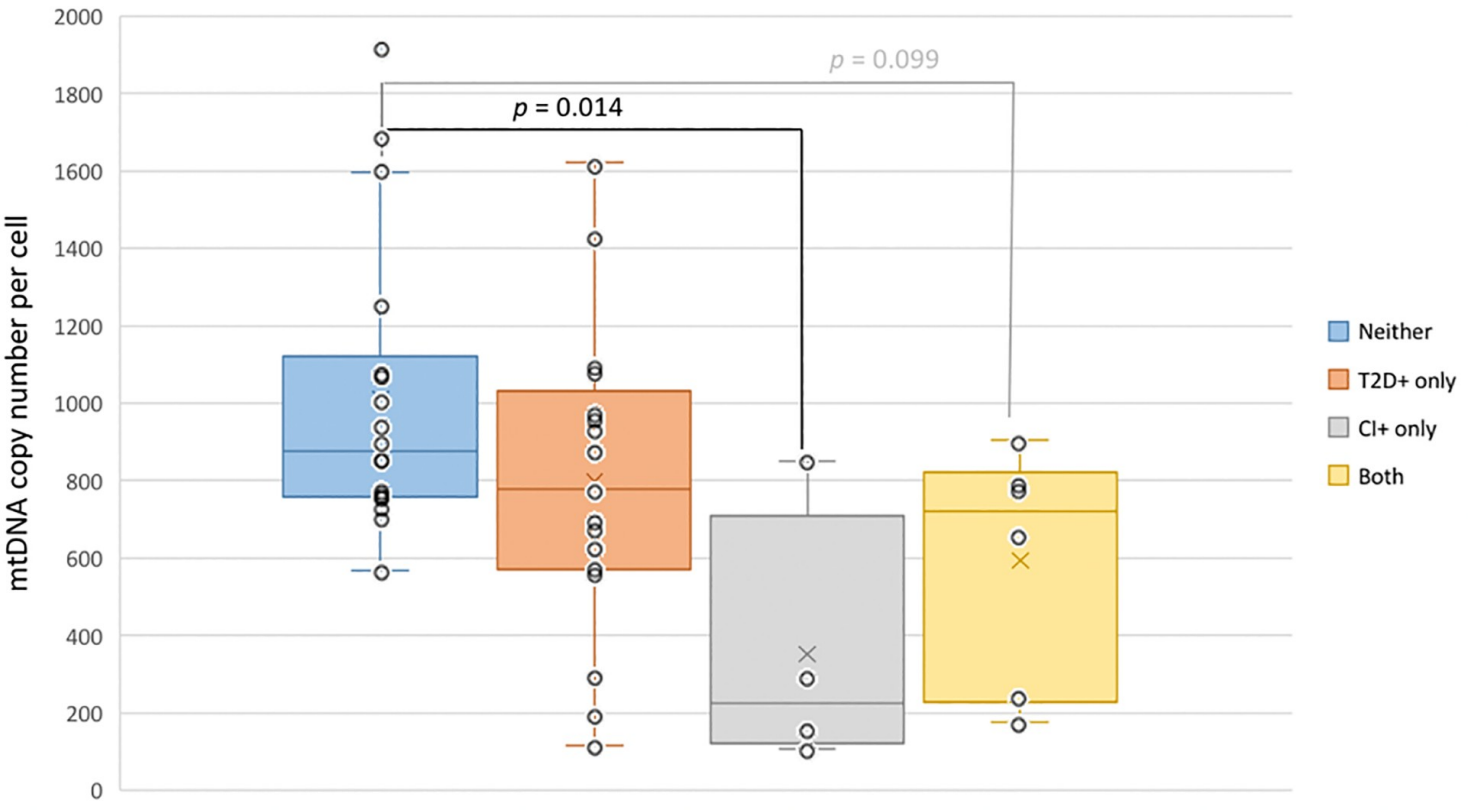
Average mitochondrial DNA copy number stratified by condition. Copy number of mitochondrial genomes is significantly lower in cognitively impaired individuals when compared to controls (ANOVA p-value = 0.009, with significant Tukey post-hoc p-value = 0.014 as shown; Kruskal-Wallis Test p-value = 0.027; respective Shapiro-Wilk p-values = 0.006, 0.880, 0.131, 0.151).

**Fig 6:**
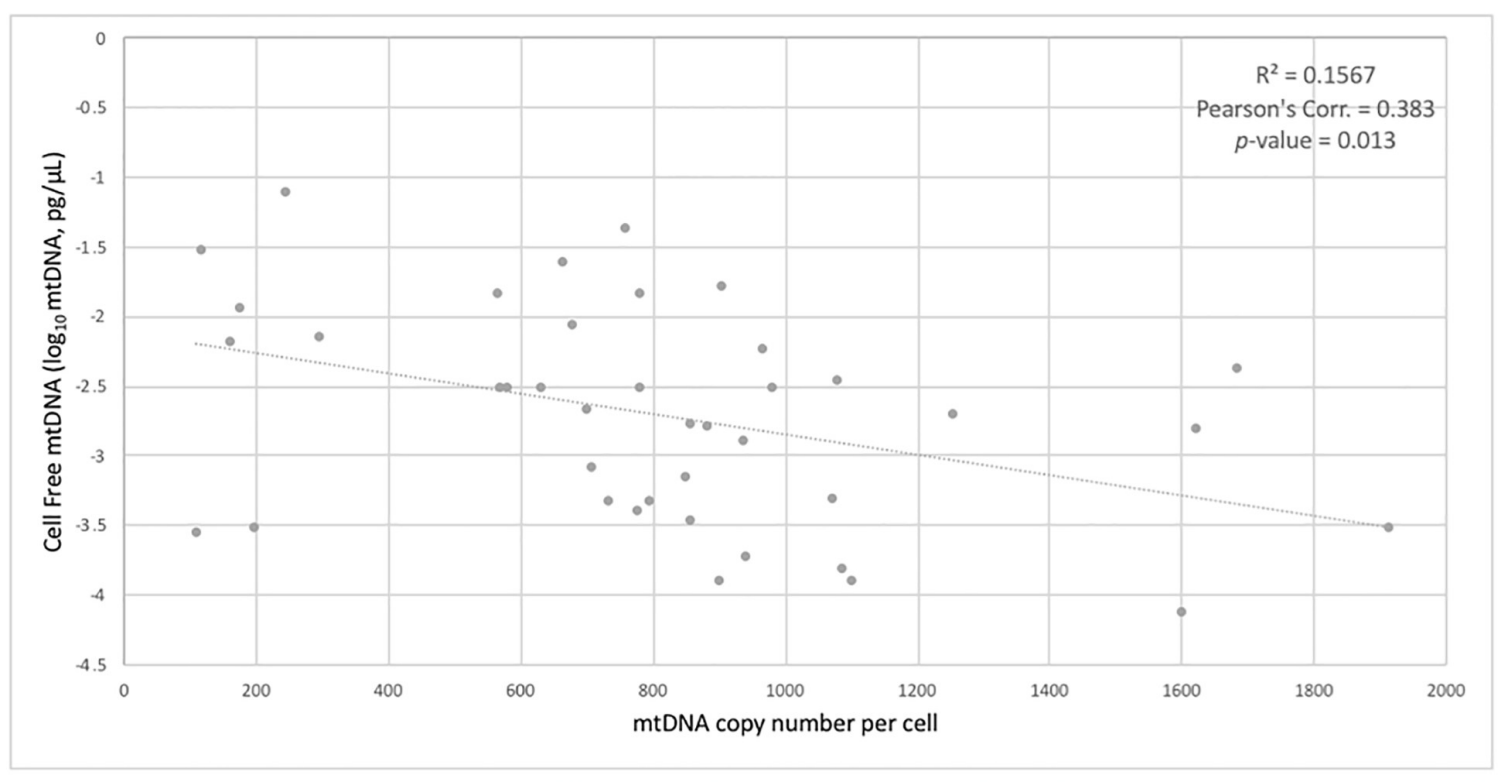
Correlations between mitochondrial indices. Pearson’s correlations showing significant relationships between cell-free mitochondrial DNA levels and mitochondrial DNA copy number (p-value = 0.013).

**Fig 7:**
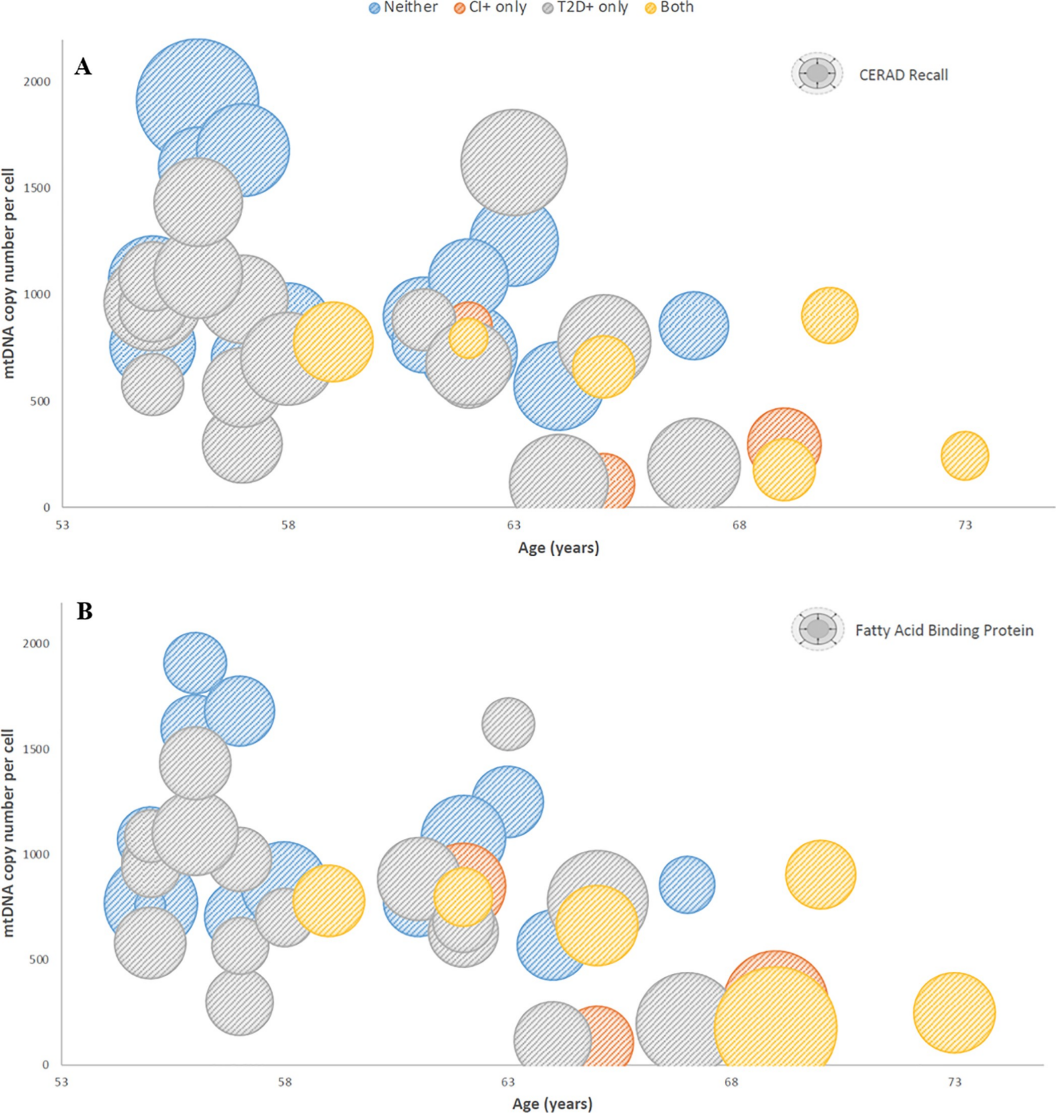
Correlations between age, mitochondrial DNA copy number, CERAD and FABP levels. Bubble charts displaying significant correlations between age, mitochondrial DNA copy number and (A) CERAD recall score [observed range 2–19, higher indicating better cognitive function] (Pearson’s corr. = 0.464, p-value = 0.001); (B) fatty acid binding protein levels (Pearson’s corr. = -0.363, p-value = 0.007) (indicated as size of bubble). Age and mtDNACN have a significant negative correlation (Pearson’s corr. = -0.288, p-value = 0.035).

When stratifying for all four conditions 1) neither disease, 2) T2D+ only, 3) CI+ only and 4) both diseases, ANOVA with Tukey post-hoc yielded a significant difference in mtDNA_CN_ between individuals with neither disease and individuals suffering only from CI (*p*-value = 0.014) (Fig 5). In addition, mtDNA_CN_ was found to significantly correlate with CFmtDNA levels (*p*-value = 0.013) (Fig 6). Mitochondrial DNA copy number values were also compared to a variety of neuropsychological variables and physiological data to determine the presence of any significant correlations. Pearson’s correlation revealed significant positive/negative relationships between mtDNA_CN_ and CERAD recall score/age, respectively (Fig 7A). A significant negative relationship was also observed between mtDNA_CN_ and fatty acid binding protein (FABP) levels (Fig 7B). Further, FABP levels were found to positively correlate with age (Fig 7B).

### Candidate gene association study

**Fig 8:**
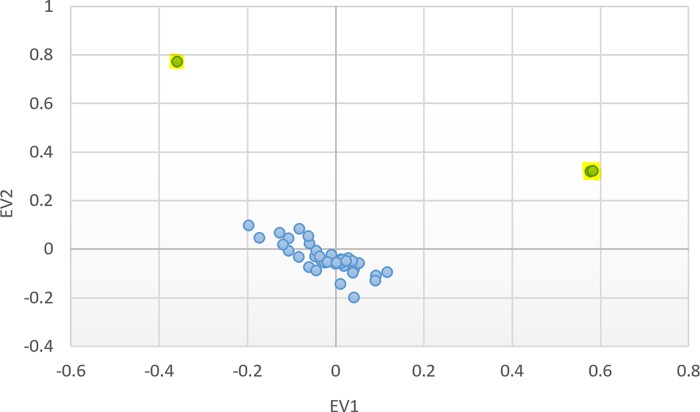
Principle Component Analysis for Population Structure. Three outliers (highlighted) were identified when plotting the first two eigenvectors from smartpca analysis. Eigenvalues for EV1 and EV2 are 1.276 and 1.164, respectively.

Dataset filtering based on individual missingness (—mind) resulted in removal of 0 individuals. SNP filtering based on missingness (—geno) resulted in removal of 26,414 markers; filtering rare SNPs based on minor allele frequency (—maf) resulted in removal of 1,306,853 markers. Hardy-Weinberg thresholding (—hwe) removed 1,082 SNPs. PCA plots of the first two eigenvectors revealed three outlying individuals, who were removed prior to subsequent analysis (Fig 8); a relatively small proportion of variance was accounted for in the first two eigenvectors, so population substructure effects are likely minimal; regardless, the EV1 and EV2 values were used as covariates in the linear model for the gene set tests.

Gene sets were established and the total number of SNPs within each gene (and 300kb surrounding window) were totaled (Table 2). While multiple testing for the total number of SNPs in each gene set was corrected for using PLINK’s permutation-based SNP-set testing protocol, additional post-hoc a correction was calculated based on the total number of gene sets using the Bonferroni method (α_Bonferroni_ = 0.05/23 genes = 0.00217). Only one of the gene-based SNP-set tests for the candidate genes reached a Bonferroni-corrected significance—*LRRK2*, leucine-rich repeat kinase 2. Two additional gene sets from the candidate gene list reached significance, if α is not corrected for multiple testing: *MFF* (mitochondrial fission factor) and *DRP1* (a.k.a. DNM1L, dynamin-1-like protein). These three genes all play a role in regulating mitochondrial fission.

**Table 2:** Candidate genes and set-based association results with mitochondrial DNA copy number.

	Candidate Gene	Chr	Gene start (hg38)	Gene end (hg38)	Total # SNPs	# Significant	# Significant after LD pruning	Set-based empirical p-value
*biogenesis*	MTOR	1	11106531	11273497	660	12	6	0.8768
	NRF1	7	129611702	129757082	283	14	10	0.1205
	NRF2	21	177230303	177265131	189	8	7	0.06159
	PPARGC1A	4	23792021	24472829	746	14	10	0.9094
	SIRT1	10	67884669	67918390	194	10	4	0.6168
	TFAM	10	58385143	58399230	177	21	7	0.5135
*fission*	DNM1L	12	32679200	32745650	156	2	2	0.006999
	FIS1	7	101239612	101245090	287	12	10	0.9075
	HTT	4	3074681	3243960	188	0	0	1
	LRRK2	12	40224890	40369285	384	22	10	0.0003 **
	MFF	2	227325151	227357836	484	16	10	0.0115
	MIEF1	22	39500100	39518134	285	10	9	0.7863
	MIEF2	17	18260534	18265790	144	0	0	1
	PLD6	17	17200990	17206905	289	14	10	0.1392
*fusion*	MFN1	3	179347692	179393226	244	16	9	0.3213
	MFN2	1	11980181	12013515	726	32	8	0.1745
	OPA1	3	193593144	193697811	442	14	10	0.07049
*mitophagy*	BAX	19	48954825	48961798	350	1	1	0.9959
	BNIP3L	8	66985238	67008877	75	1	1	0.1384
	DAPK1	9	87497228	87708634	557	18	10	0.5024
	NDP52	17	48830988	48865245	304	21	9	0.5701
	PINK1	1	20633455	20651511	366	5	4	0.5209
	ULK1	12	131894734	131923162	286	14	8	0.5469

**p-value < α_Bonferroni,_ 0.00217; multiple testing for total number of SNPs in each gene set is corrected for using PLINK permutation-based SNP set testing protocol; additional post-hoc α correction is required only for the total number of sets (*i*.*e*., 23 candidate genes).

**Fig 9:**
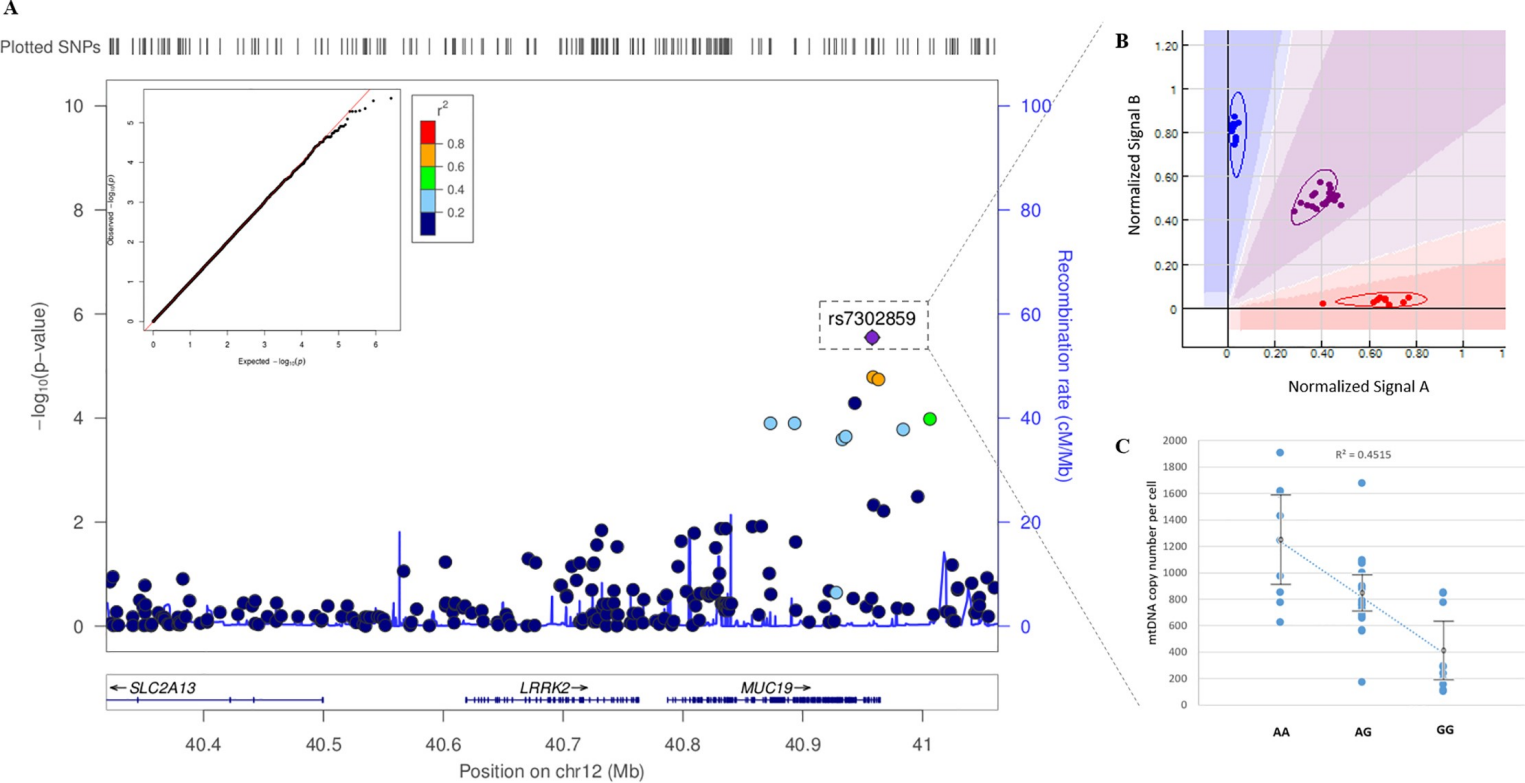
Local plot of association results. (A) Regional SNP-based association results for LRRK2 (LocusZoom). The nest Q-Q plot for single SNP associations (qqman R package) conforms to expectation and does not exhibit signs of genomic inflation. The SNP with the lowest p-value associated with mitochondrial copy number is rs7302859, and the cut-away panel (B) illustrates appropriately called genotype clusters (Genome Studio 2.0, Illumina). Eight additional SNPs in LD (as indicated by points colored according to the r2 key) with the primary SNP also seen within the signal. (C) mtDNA copy number was found to significantly differ between genotype groups after covariate analysis of age, EV1 and EV2 (*p* = 0.008 for genotypic difference, followed by *p* = 0.003 for age; EV1 and EV2 were non-significant).

The primary driver SNP for the gene-based association result in the *LRRK2* SNP set is rs7302859, as seen in the regional association plot (Fig 9A). This plot shows the–log_10_ (*p*-value) for all single SNP tests for the *LRRK2* gene. The regional signal is actually within *MUC19* (mucin 19), a nearby gene. Eight additional SNPs in linkage with the primary SNP rise above the basal noise (Fig 9A, indicated by color-filled data points, according to the r2 key). This linkage provides additional strength for the association signal. Quality assessment of genotype calling/clustering algorithm via manual inspection each SNP clearly shows tight clusters (shown in the cutaway in Fig 9A), and all individuals were called with high confidence. The Q-Q plot of observed vs. expected p-values confirms the absence of genomic inflation (Fig 9B).

## Discussion

### Cell-free mitochondrial DNA is increased with T2D and the co-occurrence of T2D and cognitive impairment

Cell-free mitochondrial DNA has been studied as a biomarker for systemic inflammation and is a hallmark of many age-related diseases. In this study, we quantified and compared levels of CFmtDNA in plasma isolated from peripheral blood. The significantly higher CFmtDNA levels observed in individuals with T2D when compared to controls (Fig 1A) were not surprising, given the inflammatory nature of the disease. Although not significant, CFmtDNA levels were also found to be elevated in individuals with cognitive impairment (Fig 1B). This may be due to cohort design (paired design was based on T2D status) as subjects were no longer age-matched when stratifying for cognitive status (*i*.*e*., the CI+ group is of more advanced age, Table 1), and CFmtDNA levels were found to significantly associate with age (Fig 3). When analyzing CFmtDNA across all four conditions, a significant difference was only observed between individuals suffering from neither disease and individuals suffering from both diseases (Fig 2), which may implicate CFmtDNA in the exacerbation of T2D-related cognitive decline. The lack of a significant difference seen between individuals with either disease alone may be resolved in future studies with increased sample size.

CFmtDNA was analyzed for correlation with neuropsychological variables and physiological data collected on the subjects. These data include various cognitive performance outcomes, routine blood analyte analysis, and targeted blood protein quantifications (based on protein panel discussed in [9]). CFmtDNA did not correlate with any of the variables analyzed (*p*>0.05), which was somewhat unexpected given previous reports of correlations between inflammatory markers in blood [45]. This observation may be due to the sample type analyzed—the protein concentrations were assessed in blood serum whereas the CFmtDNA quantifications were performed on blood plasma. Other groups have observed quantities of certain inflammatory markers to differ between plasma and serum [46].

### Mitochondrial DNA copy number is decreased with cognitive impairment

Mitochondrial DNA copy number has been implicated in both aging and age-related diseases and has been utilized as an indicator of mitochondrial biogenesis. Here, we quantified mtDNA_CN_ per cell from peripheral blood buffy coat, the leukocyte-enriched blood fraction. Significantly lower mtDNA_CN_ was observed in cognitively impaired individuals when compared to controls (Fig 4B). Since the CI- and CI+ groups are not matched for age, and mitochondrial copy number increases with age (Fig 7A and 7B), it is possible that the effect seen in Fig 4B is an age-related effect. However, when analyzing copy number across all four conditions (Fig 5), mtDNA_CN_ was found to be significantly decreased in those with CI only (CI+) when compared to controls, and age does not explain the effect (*i*.*e*., the “Both” group is slightly older than the CI+ only). The same trend was also observed in diabetic individuals, but it did not reach significance (Fig 4A). It is possible that the larger effect size in mtDNA_CN_ observed for the cognitively impaired points to alteration(s) in mitochondrial regulation within the cell. By contrast, the T2D-effect seen in CFmtDNA (Figs 1 and 2), which is extracellular, is perhaps increased as a by-product of mitochondrial dysfunction. Although the mechanism for copy number regulation is relatively unclear, there may be underlying genetic factors conferring increased risk for CI and/or declines in mtDNA_CN_. Interestingly, mtDNA_CN_ was also found to significantly correlate with CFmtDNA (Fig 6), suggesting a unique biological relationship between the two mitochondrial indices.

In addition, when analyzing both neuropsychological variables and physiological data, suggestive correlations were observed among mitochondrial copy number, age and CERAD recall score (Fig 7A) and fatty acid binding protein levels (Fig 7B). CERAD recall is a sub-set of a battery of neuropsychological tests responsible for assessing list learning and memory recall ability, used to assess cognitive function. CERAD recall score was observed to have a positive association with mtDNA_CN_, where higher copy number was associated with higher CERAD score (positively correlates with cognitive function) (Fig 7A). This may signify the potential utility of quantifying mitochondrial indices from peripheral blood as a means of detecting severity of cognitive decline. FABP levels were found to negatively correlate with mtDNA_CN_ and positively correlate with age (Fig 7B), which is concordant with previous reports by other groups [18–22, 47]. Interestingly, FABP was the protein with the largest fold-change in the previously reported blood-based predictive model for AD in Mexican American populations [9]. This suggests that mtDNA_CN_, a novel biomarker in this context, may enhance existing predictive models.

### Variants in LRRK2/MUC19 may play a role in regulating mtDNA_CN_

Although the mechanisms for regulating mtDNA_CN_ are unclear, our candidate gene-set analyses point to proteins that may be involved in the control of mitochondrial copy number. Specifically, the only SNP-set with a significant association with mtDNA_CN_ following Bonferroni correction, was in the *LRRK2* region (Table 2). *LRRK2* encodes the cytoplasmic protein leucine-rich repeat kinase 2, which is capable of interacting with PINK1 and Parkin in the autophagy process [48]. While specific mutations in *LRRK2* are causal of autosomal dominant forms of late-onset parkinsonism, its expression patterns and role in mitochondrial regulation has been implicated in the context of other complex conditions as well. Overexpression of LRRK2 in diabetes has been demonstrated in a rat model, leading to greater fragmentation of mitochondrial organelles [49]. It has also been shown to interact with DRP-1 (DNM1L), dynamin-related protein-1, in regulating mitochondrial dynamics [50]. For example, in Parkinson’s disease, wild type LRRK2 expression has been observed to cause mitochondrial fragmentation and increased levels of DRP-1 [50]. DRP-1 is known mainly for the role it plays in mitochondrial and peroxisomal fission. SNPs within *DRP-1* were found to have an association with mtDNA_CN_ (result did not reach significance after Bonferroni correction) (Table 2), which may further support the potential role of fission proteins in regulating mtDNA_CN._ Interestingly, DRP-1 has been implicated in T2D, whereby imbalances in DRP-1 signaling have been observed in diabetic mice [51]. Additionally, DRP-1 is known to interact with amyloid beta, with increasing rate of interaction as AD pathology progresses and has been suggested as a potential therapeutic target for AD [52]. With this, it was not surprising to also see a suggestive association between mtDNA_CN_ and SNPs within *MFF* (Table 2), mitochondrial fission factor, known to encode a receptor on the OMM that binds DRP-1 [53].

*MUC19*, the neighboring gene where the primary signal was actually observed (Fig 9A), encodes a member of the heavily O-glycosylated mucin protein family (GenBank Gene ID: 283463). The most significantly associated SNP, rs7302859, is located between exon 166 and 167 of *MUC19*; the SNP is also within 5kb of a H3K27Ac signature (from ENCODE data on GM12878 B-lymphocyte expression studies). Variants in both *MUC19* and *LRRK2* have been reported together in multiple GWAS investigating Crohn’s disease [54], ankylosing spondylitis [55], and autoimmune thyroid disease [56]. This region is often referred to as the *LRRK2*/*MUC19* locus due to shared association signals. The biological significance of this association is unclear, however, its proximity to a putative enhancer domain may suggest an uncharacterized regulatory role for variants through the region and may include modulation of mitochondrial function/biogenesis. Of course, the association reported here should be replicated in an independent dataset, especially given the small sample size and relatively modest effect size. Follow-up fine mapping may also help clarify if this signal is associated with copy number due to linkage disequilibrium with *LRRK2* versus an independent effect of variation in *MUC19*.

## Concluding remarks

**Fig 10:**
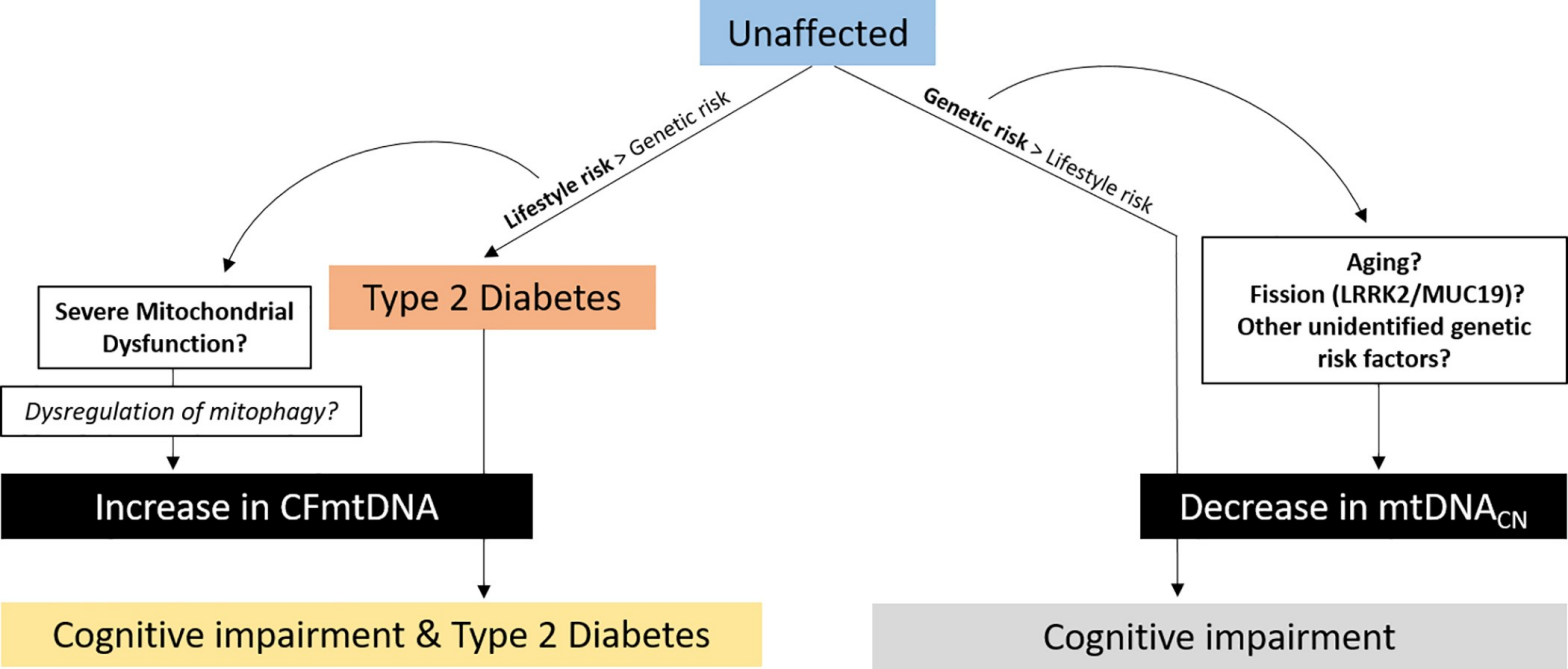
Hypothetical schematic for biological context of results. Type 2 diabetes is often considered to be a disease of lifestyle, while cognitive impairment (*e*.*g*., Alzheimer’s Disease) is presumed to be more founded in genetics, based on higher estimates of heritability. Increased CFmtDNA observed in subjects with both CI and T2D may be the result of lifestyle-sourced mitochondrial dysfunction, ultimately impacting mitophagy and resulting in release of mitochondrial components extracellularly. Decrease mtDNA_CN_ was a more prominent feature in subjects with CI only. Genetic risk factors affecting mitochondrial dynamics and/or aging may underlie this trend.

The difference in effect observed for these mitochondrial indices when comparing T2D and CI may be due to their dissimilar risks, as T2D is often regarded as a disease of lifestyle and AD has a larger presumed genetic component. Fig 10 provides a hypothetical schematic which summarizes the findings from this study. The data provided here indicate that CFmtDNA and mtDNA_CN_ are likely indicative of two different mitochondrially-sourced pathogeneses, both ultimately resulting in cognitive impairment. CFmtDNA appears to be a T2D-centric phenotype, which is exacerbated with advancing age, as seen in individuals with both T2D and CI. We posit that systemic, metabolic challenges in the confluence of diabetes and age result in T2D-related cognitive decline, and the release of mtDNA into the extracellular space may be indicative of this progression. On the other hand, mtDNA_CN_ effects are more pronounced in individuals who are aged and have CI in the absence of T2D. This perhaps indicates that unknown genetic and/or lifestyle-based risk factors for advancing age or altered mitochondrial dynamics (*i*.*e*., variants in *LRRK2/MUC19*?) may contribute to decreased cognitive function, as indicated by depressed mtDNA_CN_. However, it should be noted that diabetes medications such as Metformin are known to target mitochondrial function; these effects were not accounted for here and may be a confounding factor in interpretation of this study. Follow-up studies to evaluate the impact of altered mitochondrial indices (*i*.*e*., CFmtDNA and mtDNA_CN_) on biological function will help to paint a more complete picture of the role mitochondria and mitochondrial function are playing in the pathophysiology of these two age-related diseases.

## Supporting information

S1 File Candidate Gene Study ped. (PED)

S2 File Candidate Gene Study map. (MAP)

S3 File Minimal dataset. (XLSX)
